# Antibacterial and Antifungal Properties of Silver Nanoparticles—Effect of a Surface-Stabilizing Agent

**DOI:** 10.3390/biom11101481

**Published:** 2021-10-07

**Authors:** Agnieszka Gibała, Paulina Żeliszewska, Tomasz Gosiewski, Agnieszka Krawczyk, Dorota Duraczyńska, Joanna Szaleniec, Maciej Szaleniec, Magdalena Oćwieja

**Affiliations:** 1 Department of Molecular Medical Microbiology, Chair of Microbiology, Faculty of Medicine, Jagiellonian University Medical College, Czysta 18, 31-12 Krakow, Poland; tomasz.gosiewski@uj.edu.pl (T.G.); agnieszka.krawczyk@doctoral.uj.edu.pl (A.K.); 2 Jerzy Haber Institute of Catalysis and Surface Chemistry, Polish Academy of Sciences, Niezapominajek 8, 30-239 Krakow, Poland; paulina.zeliszewska@ikifp.edu.pl (P.Ż.); dorota.duraczynska@ikifp.edu.pl (D.D.); maciej.szaleniec@ikifp.edu.pl (M.S.); magdalena.ocwieja@ikifp.edu.pl (M.O.); 3 Department of Otolaryngology, Faculty of Medicine, Jagiellonian University Medical College, Jakubowskiego 2, 30-688 Krakow, Poland; joanna.szaleniec@uj.edu.pl

**Keywords:** silver nanoparticles, biocidal properties, surface properties, Gram-negative bacteria, Gram-positive bacteria, pathogenic fungi, *Escherichia coli*, *Staphyloccus aureus*, *Candida albicans*, minimum inhibitory concentration (MIC), minimum bactericidal concentration (MBC)

## Abstract

The biocidal properties of silver nanoparticles (AgNPs) prepared with the use of biologically active compounds seem to be especially significant for biological and medical application. Therefore, the aim of this research was to determine and compare the antibacterial and fungicidal properties of fifteen types of AgNPs. The main hypothesis was that the biological activity of AgNPs characterized by comparable size distributions, shapes, and ion release profiles is dependent on the properties of stabilizing agent molecules adsorbed on their surfaces. *Escherichia coli* and *Staphylococcus aureus* were selected as models of two types of bacterial cells. *Candida albicans* was selected for the research as a representative type of eukaryotic microorganism. The conducted studies reveal that larger AgNPs can be more biocidal than smaller ones. It was found that positively charged arginine-stabilized AgNPs (ARGSBAgNPs) were the most biocidal among all studied nanoparticles. The strongest fungicidal properties were detected for negatively charged EGCGAgNPs obtained using (−)-epigallocatechin gallate (EGCG). It was concluded that, by applying a specific stabilizing agent, one can tune the selectivity of AgNP toxicity towards desired pathogens. It was established that *E. coli* was more sensitive to AgNP exposure than *S. aureus* regardless of AgNP size and surface properties.

## 1. Introduction

Silver is widely known for its biocidal properties. It exhibits bactericidal, fungicidal, and virucidal properties regardless of the form in which it occurs, e.g., silver ions (Ag^+^), silver complexes, and metallic silver (Ag^0^), including silver nanoparticles (AgNPs). Due to their high biological activity, silver compounds are broadly applied, especially in diverse branches of biology and medicine. Silver-containing materials are used, for example, to reduce infection in burn treatment and arthroplasty and to prevent bacteria colonization on prostheses, catheters, vascular grafts, dental materials, stainless steel materials, and human skin [1,2,3].

The biological efficiency of silver in the form of AgNPs is higher than that of conventional (bulk) silver due to the high surface-to-volume ratio [4]. There is no doubt that silver ions and AgNPs exhibit biocidal activity towards prokaryotic and eukaryotic cells [5,6]. It is generally accepted that silver ions and AgNPs are more biocidal towards prokaryotes than eukaryotic cells, leading to a therapeutic window where mammalian tissue is not harmed but where bacteria are killed [7]. It is worth emphasizing that AgNPs are less chemically reactive than silver ions, which interact with a variety of biomolecules within a cell such as nucleic acids, cell wall components, sulfhydryl groups of metabolic enzymes, and sulfur-containing cell components such as glutathione [8,9]. However, it should also be remembered that AgNPs are a source of silver ions. AgNPs are susceptible to oxidative dissolution, which leads to the continuous release of silver ions [10]. The rate of silver ion leaching from AgNPs depends on external conditions and physicochemical properties of AgNPs [11,12,13,14].

The possibility of shaping AgNPs’ biological activity by the control of their physicochemical properties is another advantage that induces their wide application [15,16]. It was proven that smaller AgNPs are more toxic than larger ones [17,18,19]. This dependence is correlated to two important factors. Firstly, AgNPs characterized by smaller sizes more efficiently enter cells and diverse microorganisms [20,21,22]. Additionally, smaller AgNPs are more sensitive to oxidative dissolution and, as a result, generate more silver ions than larger AgNPs in shorter periods of time [13,23].

The shape of AgNPs is the next pivotal factor affecting biocidal properties [24]. Pal et al. [25] studied the antibacterial activity of sphere-shaped, rod-shaped, and truncated triangular AgNPs towards *Escherichia coli*. The results of the conducted works revealed that truncated triangular AgNPs exhibited the highest biocidal activity followed by nanospheres and nanorods.

The formation of anisotropic AgNPs is induced and directed by specific, high-molar-mass molecules of surfactants or polymers [26]. Often, shape-controlling molecules are deposited on the surfaces of AgNPs and, similar to silver, they exhibit biological activity. For this reason, the surface chemistry of AgNPs is a crucial parameter determining the biocidal activity of the whole nanometric system [16].

Usually, AgNPs stabilized by inorganic anions are negatively charged. In turn, AgNPs covered by organic compounds having moieties capable of protonation and deprotonation can be negatively or positively charged [27]. As various scientific reports have shown, the surface charge of AgNPs affects the formation of a protein corona around nanoparticles [28] as well as their further interactions with the cell membrane [29,30,31].

Based on this literature, one can conclude that chemicals used as reducing and stabilizing agents of AgNPs are major factors modeling biological activity [16]. The impact of reducing and stabilizing agents on AgNP toxicity can be indirect, e.g., if they amplify silver ion release [32] or intensify the penetration of AgNPs through biological membranes [33]. On the other hand, stabilizing agents adsorbed on the surfaces of AgNPs can also act directly on cells and microorganisms. Hence, the use of AgNP-stabilizing agents exhibiting biocidal properties can create synergistic effects and enhance the toxicity of silver [33,34]. Nowadays, the attention of scientists is focused on the development of methods of preparation of AgNPs characterized by controlled activity towards pathogens [35] and tumor cells [36]. The selective action of AgNPs towards normal and pathogenic cells as well as towards beneficial and harmful microorganisms is highly desired. Previously, it was postulated that AgNPs obtained in green synthesis processes [37,38] with the use of plant extracts [39] are highly toxic for pathogens and practically inert for normal cells [40]. More sophisticated studies revealed that the desired selectivity of AgNPs can be achieved only by directed functionalization of AgNP surfaces conducted with the use of specific bioactive molecules [36].

Irrespective of these facts, one can notice that usually the functionalization of AgNPs is carried out based on well-defined AgNPs obtained in conventional synthesis processes, e.g., the citrate process [41] or Turkevich’s method [42]. Current literature reports also showed that biocidal properties of AgNPs obtained by conventional methods of synthesis are rarely determined with respect to Gram-positive and Gram-negative bacteria as well as fungi in concurrent and comparative tests. In this way, our knowledge about the biocidal properties of AgNPs is limited. The biological activity of AgNPs obtained in a given synthesis protocol is evaluated by independent scientific groups based on diverse research protocols. This approach can lead to differences in the obtained results regarding, e.g., values of minimum inhibitory concentration (MIC) and minimum bactericidal concentration (MBC). Hence, there is a demand for the continuation of research on biocidal properties of AgNPs stabilized by diverse low-molecular-mass compounds and obtained using simple and efficient chemical reduction methods.

Taking into account the aforementioned issues, the aim of this research was to determine and compare biocidal properties of several types of AgNPs obtained with the use of available chemicals of well-documented biological activity. The main hypothesis was that the biological activity of AgNPs characterized by comparable size distributions, shapes, and ion release profiles is dependent on the properties of the stabilizing agent molecules adsorbed on their surfaces. It was assumed that the biocidal activity of AgNPs will be mainly tuned by the presence of stabilizing agent molecules whereas the role of the surface charge generated by these molecules will be a secondary factor. *E. coli* and *S. aureus* were applied as models of two types of bacterial cells respectively Gram-negative and Gram-positive for the evaluation of bactericidal properties of the synthesized AgNPs. *Candida albicans* was selected for the research as a representative type of eukaryotic microorganism that may cause fungal infections in humans. The biocidal activity of diverse types of AgNPs towards these pathogens was assessed based on the determined values of MIC and MBC.

## 2. Materials and Methods

### 2.1. Reagents

All chemicals used for the preparation of AgNPs were supplied by Sigma-Aldrich. These chemicals were of analytical grade and used without further purification. Ultrapure water (Milli-Q water) of conductivity 0.06 μS cm^−1^ was obtained using a Milli-Q Elix&Simplicity 185 purification system (Millipore SA, Molsheim, France).

### 2.2. Microorganisms

American Type Culture Collection (ATCC) delivered the prokaryotic bacteria strains *Escherichia coli* (ATCC 25922) and *Staphylococcus aureus* (ATCC 29213) as well as *Candida albicans* (ATCC 10231), which is a representative eukaryotic fungal pathogen.

### 2.3. Synthesis of AgNPs

Each type of AgNP was prepared by a chemical reduction of silver ions delivered in the form of silver nitrate by selected reducing agents (Table 1). In the case of reducing agents that do not exhibit stabilizing properties, additional chemicals playing this role were used. The AgNPs were prepared in the form of aqueous suspensions. Detailed preparation procedures are described in the Supplementary Materials. For convenience, the AgNPs were marked using the first letters of names of chemicals used during their preparation.

Each AgNP suspension was purified from low-molecular-mass impurities via the ultrafiltration method. For this purpose, the suspensions were washed with Milli-Q water using an Amicon^®^ filtration cell (model 8400) equipped with membranes made of regenerated cellulose of a nominal molecular weight limit of 100 kDa. The purification process was carried out until the conductivity of the effluents stabilized at 20 μS cm^−1^ and the pH attained a value of ca. 5.8–6.1. In the case of AgNPs obtained using cysteine (CYSSBAgNPs), the purification process was conducted using a 0.1 mM nitric acid solution [27]. The stock suspensions were stored in a refrigerator at a temperature of 4 °C.

### 2.4. Physicochemical Characteristics of AgNPs

An ADMA500 M densitometer (Anton Paar, Graz, Austria) was used to measure the density of stock suspensions of AgNPs and obtained effluents. The procedure described previously [43] enabled the determination of the mass concentration of AgNPs in the stock suspensions based on the density measurements and the specific density of silver, which is equal to 10.49 g cm^−3^. A Seven Compact TM pH/ionometer (Mettler Toledo, Columbus, OH, USA) equipped with a perfectIONTM silver/sulfide electrode was used to evaluate the oxidative dissolution of AgNPs and the concentration of released silver ions present in the effluents. To do this, the stock suspensions of a controlled concentration were filtered using a regenerated cellulose membrane (Millipore, nominal molecular weight limit 30 kDa) in order to separate the AgNPs from leached silver ions. Afterward, the concentration of silver ions in the effluents was determined under selected conditions: temperature, pH, and concentration of dissolved oxygen (DO), which was measured using a COG-1t oxygen probe connected to the CPO-505 oxygenmeter (Elmetron, Zabrze, Poland).

A UV-2600 spectrometer (Shimadzu, Kyoto, Japan) was used to determine the optical properties of AgNPs by examining the extinction spectra of their suspensions. In order to perform the research, AgNPs and the culture medium were dispersed in an aqueous suspension with specific parameters (pH, ionic strength, and temperature).

An AJEOL JSM-7500F electron microscope working in the transmission mode (TEM) was used to evaluate the morphology and size distribution of AgNPs. The obtained micrographs were analyzed with the use of MultiScan software (Computer scanning system). The histograms were generated from the analysis of no less than 500 AgNPs.

A Zetasizer Nano ZS instrument (Malvern Instruments, Malvern, UK) was used to assess the stability of AgNPs in the suspension under specific pH and ionic strength conditions and at the temperature of 37 °C through measurements of their diffusion coefficients (*D*) and electrophoretic mobility (*μ*_e_).

### 2.5. Exposure of Microorganisms to AgNPs

The bacteria and fungi were inoculated on Columbia agar with 5% Sheep Blood (Oxoid, Basingstoke, UK), incubated for 18 h at 36 °C, to obtain pure cultures. Afterward, 0.5 optical density (OD) on the McFarland scale was made to receive a 1.5 × 108 colony forming unit (CFU) mL^−1^ solution, then the cell suspension was diluted to 106 CFU mL^−1^. The prepared suspension was used for further studies, including the determination of MIC and MBC.

To establish the values of MIC, the suspension was inoculated on the surface of Mueller–Hinton agar (Oxoid) using a sterile cotton swab (Oxoid). Next, the surface of the agar was punched with wells using sterilized pipette tips with a diameter suitable for a 200 μL pipette. These wells were filled with an equal volume (100 μL) of the AgNP suspensions of concentration ranging from 5 to 100 mg L^−1^ (in triplicate for each concentration). In subsequent wells, the AgNP concentration was increased by 5 mg L^−1^. The well filled with pure medium (AgNP concentration equal to 0 mg L^−1^) was used as a control sample. After a period of pre-incubation (4 h at 4 °C), the inoculated plates were incubated at 37 °C over 18 h. MIC was determined as the lowest concentration of AgNP suspensions for which the zone of inhibition of microorganisms around the well was still observed.

In the case of MBC determination, the strain suspension (106 CFU mL^−1^ in liquid Mueller–Hinton broth (Merck) medium) with a series of NP concentrations from 0 to 100 mg L^−1^ (increasing in 5 mg L^−1^ steps) was incubated in the 96-well plate (Costar) for 18 h at 37 °C (the experiment was carried out in triplicate). Afterward, the samples were placed on Mueller–Hinton agar (Oxoid) and incubated at the temperature of 37 °C for 18 h. MBC was determined as the lowest concentration of AgNPs in the dilution series for which no microbial colonies were observed on the agar plate.

### 2.6. Statistical Analysis

The biological experiments were carried out in triplicate and repeated threefold independently. The results are expressed as the mean ± standard deviation. Statistical analysis was performed using single-factor analysis of variance to determine the equality of population means. Student’s *t*-tests were performed between populations of interest, with *p* < 0.05 considered statistically significant.

## 3. Results and Discussion

Fifteen types of AgNPs were synthesized in the form of aqueous suspensions using selected organic and inorganic low-molecular-mass compounds. The amounts of reagents were selected intentionally to obtain 13 types of AgNPs of comparable size distribution. Moreover, two couples of AgNPs characterized by comparable surface properties and diverse sizes were prepared. The AgNP suspensions were purified from unreacted impurities using the filtration method. The application of the purification process allowed us to obtain AgNP suspensions of comparable pH and ionic strength. The potentiometric electroanalysis involving the application of an ion-selective electrode did not reveal the presence of silver ions in the effluents collected after the last stage of the purification process. The lack of silver ions in the effluents, being dispersive media for the AgNPs, confirmed that at the beginning of experiments the suspensions contained only one form of silver, namely nanoparticles (NPs).

The first six types of AgNPs (Table 1) were prepared using sodium borohydride (NaBH_4_, SB) which is a well-known strong reducing agent widely applied for the preparation of diverse types of metal nanoparticles (MeNPs) [44,45]. Despite this fact, SB is a poor stabilizing agent and the preparation of stable MeNPs requires the presence of other efficient capping molecules. Usually, the formation of AgNPs initiated by SB occurs under acidic conditions, which are beneficial for a surface modification of newly formed nanoparticles by amino acids [46]. Moreover, a decreased pH of the reaction mixture is suitable for the preparation of positively charged AgNPs stabilized by diverse molecules having protonated moieties. Considering these issues, two aminothiols—cysteamine hydrochloride (CH) and cysteine (CYS), which can be considered as carboxylated cysteamine—were applied for AgNP stabilization. Among other amino acids, lysine (LYZ) and arginine (ARG) were selected for these studies.

The next three types of AgNPs were formed using trisodium citrate (HOC(COONa)(CH_2_COONa)_2_, TC). In the first approach, TC appeared together with SB (Table 1). The combination of SB with TC is described in the literature for the preparation of relatively small and stable MeNPs [41]. At elevated temperatures, TC is transformed into the active form, which enables the formation of larger AgNPs [45]. At ambient temperatures, TC does not exhibit reducing properties but its presence during AgNP preparation is advantageous with respect to their stabilization. This fact was exploited during the AgNP synthesis with the use of ascorbic acid (AA) being also a common antioxidant.

Alkaline conditions and four other recognized antioxidants were applied for the synthesis of the following AgNPs (Table 1). Gallic acid (GA, also known as 3,4,5-trihydroxybenzoic acid) is a simple polyphenol playing the dual role of a reducing and stabilizing agent in the synthesis of MeNPs of different sizes [47]. Similar features are exhibited by (−)-epicatechin-3-gallate (EGCG), which is an ester of GA and epigallocatechin [48]. Tannic acid (TA) was the second derivative of GA used for the preparation of AgNPs.

The chemical structure of tannic acid consists of a hepta- to octa-galloyl-β-D-glucose in which, on average, two to three additional galloyl groups are esterified to a pre-existing β-1,2,3,4,6-pentagalloyl-D-glucose core [49]. In one AgNP synthesis, GA was combined with 1,3,7-trimethylxanthine, which is known as caffeine (CAF) and does not exhibit reducing properties but is able to interact efficiently with MeNP surfaces [50].

One type of AgNP was prepared using glucose (GL), which was related to the fact that TA is a derivative of this monosaccharide and GA. Moreover, it was proven that the implementation of saccharides enables the production of AgNPs with interesting antibacterial properties [2].

Selected inorganic compounds were involved in the preparation of the last two types of AgNPs (Table 1). The reduction of silver ions by hydroxylamine hydrochloride (HH), conducted under alkaline conditions and according to the protocol developed by Leopold and Lendl [51], was applied for the synthesis of HHAgNPs. Sodium hypophosphite (SH) is barely known as a reagent used in the preparation of MeNPs. Nevertheless, it was proven that its combination with sodium hexametaphosphate (SH) allows for the production of stable AgNPs of enhanced biocidal activity [33,52].

**Table 1:** Types of silver nanoparticles (AgNPs) obtained using selected reagents and described reaction conditions.

Symbol	Reducing Agent	Stabilizing Agent	T (°C)	pH	Agent for pH Adjustment	Ref.
CHSB1AgNPs	sodium borohydride (SB)	cysteamine hydrochloride (CH)	20	5.2	-	[53]
CHSB2AgNPs	sodium borohydride (SB)	cysteamine hydrochloride (CH)	20	5.3	-	[53]
CYSSBAgNPs	sodium borohydride (SB)	cysteine (CYS)	20	3.4	-	[27,54]
LYZSBAgNPs	sodium borohydride (SB)	lysine (LYZ)	20	3.7	-	-
ARGSBAgNPs	sodium borohydride (SB)	arginine (ARG)	20	3.4	-	-
TCSBAgNPs	sodium borohydride (SB)	trisodium citrate (TC)	20	7.9	-	[41]
TCAgNPs	trisodium citrate (TC)		88	9.1	-	[42]
TCAAAgNPs	ascorbic acid (AA)	trisodium citrate (TC)	25	9.5	aq. ammonia	-
GAAgNPs	gallic acid (GA)		25	8.8	aq. ammonia	[55,56]
EGCGAgNPs	(−)-epigallocatechin gallate (EGCG)		25	8.9	aq. ammonia	[50]
TAAgNPs	tannic acid (TA)		25	8.5	aq. ammonia	[49]
CFGAAgNPs	gallic acid (GA)	caffeine (CF)	25	8.8	aq. ammonia	[50]
GLAgNPs	D-glucose		25	5.3	aq. ammonia	[57]
HHAgNPs	hydroxylamine hydrochloride (HH)		25	10.5	sodium hydroxide	[51]
SHSHAgNPs	sodium hypophosphite (SH)	sodium hexameta-phosphate (SH)	40	2.2	sulfuric acid	[33,52,58]

Blue—AgNPs obtained using sodium borohydride (SB); yellow/orange—AgNPs obtained using trisodium citrate (TC); green—AgNPs obtained using selected antioxidants; grey—AgNPs obtained using glucose; red—AgNPs obtained using selected inorganic compounds.

Purified AgNP suspensions were thoroughly characterized using diverse experimental methods. At the first stage of studies, the mass concentration of AgNPs dispersed in the stock suspensions was determined based on the density measurements described in detail in previous works [43]. It was established that the AgNP concentration varied between 120 and 200 mg L^−1^. Therefore, the stock suspensions were diluted to an equal concentration of 100 mg L^−1^ using Milli-Q water. The pH of diluted AgNP suspensions ranged from 5.6 to 6.1. An exception was the CYSSBAgNP suspension, which was purified using diluted nitric acid. In this case, it was impossible to adjust the pH to the value of 5.6–6.1 because, in this range, CYSSBAgNPs were unstable due to the close occurrence of the isoelectric point [27].

**Figure 1:**
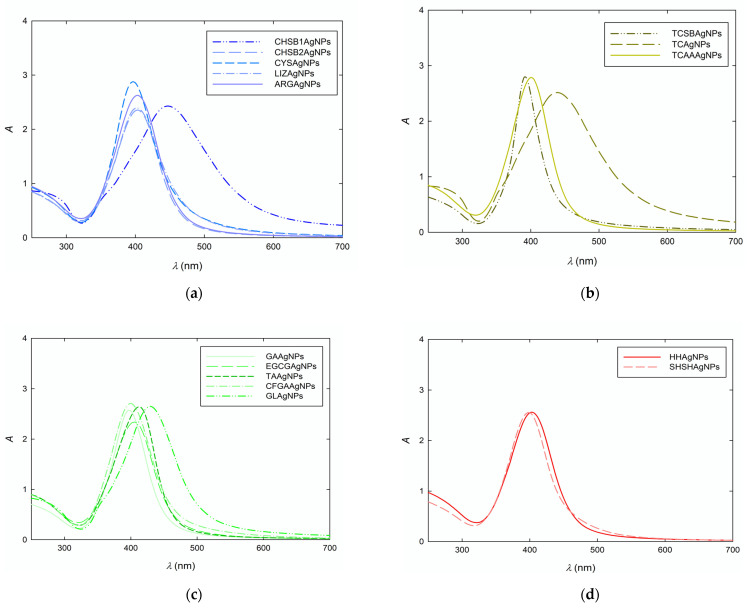
Extinction spectra of diluted AgNP suspensions prepared with the use of (**a**) sodium borohydride (SB); (**b**) trisodium citrate (TC); (**c**) selected antioxidants and glucose (GL); (**d**) inorganic compounds.

The extinction spectra of the AgNP suspensions were recorded to confirm the preparation of plasmonic nanoparticles. As is well-known from numerous literature reports, the occurrence of localized surface plasmon resonance (LSPR) induces the appearance of characteristic bands in the UV-vis spectra of AgNP suspensions [59]. The number of bands and their position in the UV-vis spectrum is correlated to the size and shape of plasmonic nanoparticles. The extinction spectra of AgNP suspensions are presented in Figure 1. As can be seen, each spectrum includes one maximum absorption band. In most cases, this band appears at a wavelength of 396–412 nm. The bathochromic shifts to the values of 438 and 447 nm are noticeable only for CHSB1AgNPs and TCAgNPs. The exact values of the absorption band of each AgNP suspension are collected in Table 2. Based on these results, one can state that the suspensions contain AgNPs of spherical shapes.

The precise shape and size distribution of the synthesized AgNPs were studied with transmission electron microscopy (TEM). The obtained histograms and typical TEM micrographs presented by the AgNPs are shown in the Supplementary Materials. It was confirmed that the AgNPs exhibit a nearly spherical shape and a quite narrow size distribution. The average size of each type of AgNP, determined based on the histograms, is given in Table 2. As can be noticed, the data obtained based on TEM imaging remained in good agreement with the findings from UV-vis spectra. It was established that the average size of most AgNPs (12 types) was in the range of 10–17 nm. The size of GLAgNPs was equal to 23 ± 8 nm, whereas CHSB1AgNPs and TCAgNPs were the largest of all the AgNPs. The average size of CHSB1AgNPs and TCAgNPs was equal to 55 ± 9 nm and 57 ± 10 nm, respectively. Based on the TEM analysis, the polydispersity index (PdI) was also calculated for each type of AgNP. The PdI values are presented in Table 2.

The AgNPs were also characterized using the dynamic light scattering (DLS) technique, which allowed us to determine their diffusion coefficients in the stock suspensions at the temperature of 37 °C, which is typical for biological experiments. The obtained values (Table 2) were used to calculate the hydrodynamic diameters of AgNPs based on the Stock–Einstein equation.

The values of diffusion coefficients and hydrodynamic diameters of AgNPs are presented in Table 2. Analyzing these data, one can notice that TCAgNPs and CHSB1AgNPs were characterized by the lowest values of diffusion coefficients. It is worth mentioning that the values of hydrodynamic diameters remain in good agreement with the results gained from TEM imaging. This confirmed that each type of AgNP is stabilized by low-molar-mass compounds that create a thin stabilizing layer on the AgNP surface.

The electrophoretic mobility measurements were carried out to determine the surface properties of AgNPs dispersed in the stock suspensions. Taking into account that zeta potential is a much more useful parameter to present the electrokinetic properties of colloidal particles, it was calculated using Henry’s equation and the measured values of electrophoretic mobility [27]. The obtained results are presented in Table 2. As can be noticed, the first five types of AgNPs were positively charged. The zeta potential of the largest CHSB1AgNPs was the highest and equal to 70 ± 2 mV. The smaller CHSB2AgNPs were less charged and their zeta potential reached the value of 51 ± 2 mV. In turn, the zeta potential of CYSSBAgNPs at pH 4.0 was equal to 40 ± 4 mV. Previously, based on the results of studies from surface-enhanced Raman spectroscopy (SERS), it was described that protonation of the amine moiety of cysteamine and cysteine causes the MeNPs stabilized by them to have a positive charge [53]. Nevertheless, cysteamine-stabilized AgNPs are positively charged even under strongly alkaline conditions (pH 10) [53], whereas cysteine-stabilized AgNPs possess an isoelectric point at pH 5.1 [27]. Therefore, one can emphasize that CYSSBAgNPs at pH 7.4, which was applied in the biological part of the studies, were negatively charged and the value of their zeta potential dropped to −39 ± 3 mV [27].

To the best of our knowledge, the physicochemical properties of lysine- and arginine-stabilized MeNPs have not been described in the literature yet. Herein, it was found that LYZSBAgNPs were characterized by a slightly lower value of zeta potential than ARGSBAgNPs (Table 2). The data obtained from additionally conducted research reveal that these AgNPs were positively charged at pH 7.4.

The rest of investigated AgNPs were negatively charged at the pH characteristic for the stock suspension as well as at pH 7.4. It is well-known that MeNPs prepared with the use of trisodium citrate are stabilized by unreacted citrate anions [60]. Hence, TCSBAgNPs and TCAgNPs possess comparable values of zeta potential, which remain in good agreement with other scientific reports [61]. In terms of TCAAAgNPs, one can suspect that a slight decrease in the zeta potential in relation to TCSBAgNPs arises from the appearance of an oxidized form of ascorbic acid in the stabilizing layers of these nanoparticles. Previous studies carried out using SERS showed that AgNPs obtained according to a reduction procedure involving CT and AA can be stabilized by dehydroascorbic acid (DHA) molecules [52]. Recorded SERS spectra did not reveal any bands characteristic for CT and pure AA [52].

The zeta potential of other AgNPs prepared using selected common antioxidants attained highly negative values. EGCGAgNPs were characterized by the highest negative zeta potential (−61 ± 1 mV). In turn, CAFGAAgNPs exhibited the least negative zeta potential in this group of AgNPs (Table 2). It is worth recalling that SERS-aided research indicated that antioxidants belonging to the polyphenol class also are oxidized during AgNP synthesis and, for this reason, their unreacted forms do not participate in the formation of stabilizing layers of AgNPs. Silvaraman et al. [62], in discussing the tannic-acid-induced formation of AgNPs, paid attention to the oxidation of the phenolic group of GA molecules into quinone forms. Indeed, further SERS studies conducted by Barbasz et al. [50] showed that AgNPs obtained using GA are stabilized by their derivatives. It was detected that AgNP formation is combined with the opening of the GA ring and probable polymerization processes that lead to the creation of aromatic polymers [63]. Similarly, EGCG, which is an ester of GA and a catechin derivative, under alkaline conditions occurring during AgNP synthesis is oxidized [50,64,65].

CFGAAgNPs were obtained using CF as a stabilizing agent and GA as a reducing agent. The preparation method causes the AgNPs to be stabilized by unreacted CF molecules and derivatives of GA [50]. One can also expect that the presence of neutral CF molecules in the stabilizing layer is a reason for the less negative value of CFGAAgNP zeta potential in comparison with GAAgNPs (Table 2).

The zeta potential of GLAgNPs was equal to −50 ± 1 mV and was quite comparable to the value determined for TAAgNPs. It is worth mentioning that GLAgNPs were obtained by the application of a well-known protocol based on the reduction of the amine complex of silver by GL. Usually, an approach involving the application of saccharides as reducing and stabilizing agents of MeNPs is referred to as green synthesis [64]. Despite the broad range of applications of this synthesis route for the preparation of AgNPs for biological and medical purposes, little is known about the physicochemical properties of such MeNPs. It is obvious that GL is oxidized during the preparation of AgNPs. Nevertheless, to the best of our knowledge, the determination of the chemical structure of stabilizing layers of such AgNPs has not been undertaken yet. Moreover, the data about the electrokinetic properties of AgNPs are also poor and ambiguous.

HHAgNPs and SHSHAgNPs represent nanoparticles prepared using inorganic compounds. It was established that both types of AgNPs were characterized by high negative zeta potential values ranging between −57 and −55 mV (Table 2). It is worth emphasizing that the synthesis procedure involving HH was developed by Leopold and Lendl [51] as a response to the demand for highly active plasmonic substrates for SERS. Thereby, the suspensions obtained in this manner are free of organic contaminants and AgNPs.

The combination of sodium hypophosphite (SH) and sodium hexametaphosphate (SH) is not often used in the preparation of MeNPs. Nevertheless, it was proven that in this system, sodium hypophosphite (SH) reduces silver ions [66] and sodium hexametaphosphate (SH) plays the role of a stabilizing agent [33].

In the next stage of studies, the biological activity of well-defined AgNP suspensions of high purity was tested towards two reference strains of bacteria and fungi. *E. coli* was selected as a typical model strain of Gram-negative bacteria, whereas *S. aureus* represents Gram-positive bacteria. The antifungal activity of AgNPs was determined by applying eukaryotic *C. albicans*, which is probably one of the most successful opportunistic pathogens in humans [67]. The antibacterial properties of AgNPs were evaluated by determining the two parameters MIC and MBC, thus the lowest concentration of AgNPs at which 99.9% of the bacteria and fungi are killed. To establish these parameters, the standard dilution method on the solid medium described above was applied. The results of studies achieved for all investigated AgNPs and microorganisms are shown in Table 3.

The analysis of results obtained from biological studies was conducted with respect to the physicochemical properties of synthesized AgNPs. In the first approach, AgNP size aspects were taken into account. It should be mentioned that, among the 15 types of tested AgNPs, twelve exhibited a comparable size distribution with an average size range of 10–17 nm (Table 2). CHSB1AgNPs and TCAgNPs were characterized by the largest size (50 nm) among all investigated nanoparticles. Despite this fact, their effectiveness in the deactivation of bacteria and fungi was not the lowest in comparison with the data obtained for the smaller AgNPs (Table 3). The value of MBC determined for positively charged CHSB1AgNPs was equal to 45 mg L^−1^ for each type of pathogen. The positively charged CHSB2AgNPs with an average size of 12 nm gave higher values of MBC than CHSB1AgNPs, namely 75, 100, and 65 mg L^−1^ for *E. coli*, *S. aureus*, and *C. albicans*, respectively. Similar observations were established for the two types of negatively charged, citrate-stabilized AgNPs. In the case of the treatment of *E. coli*, the MBC was equal to 35 and 45 mg L^−1^ for the larger TCAgNPs and the smaller SBTCAgNPs, respectively (Table 3). TCAgNPs were also characterized by stronger fungicidal properties than the smaller SBTCAgNPs (Table 3). It is also worth mentioning that the 50-nm-sized CHSB1AgNPs and TCAgNPs turned out to be more biocidal for the investigated pathogens than the other types of smaller AgNPs. For example, the values of MBC detected for CHSB1AgNPs were noticeably lower than the ones established for the positively charged CYSSBAgNPs as well as the negatively charged TCAAAgNPs and GAAgNPs. These findings are surprising with respect to the fact that larger AgNPs are characterized by lower values of diffusion coefficients than smaller ones (Table 2). In the case of the well diffusion method, which was applied in these studies, the diffusion of AgNPs throughout the medium is one of the most important factors determining the efficiency of pathogen deactivation. Based on the values of the measured diffusion coefficient (Table 2), it was expected that the larger AgNPs would exhibit poor activity towards bacteria. On the other hand, numerous scientific works suggest that, usually, smaller AgNPs are more biocidal than larger ones. Some literature reports indicate that smaller AgNPs demonstrate better antibacterial activity than larger AgNPs [47,68,69,70,71]. For example, Martínez-Castañon et al. [47] prepared three types of spherical AgNPs with an average size of 7, 29, and 89 nm using GA. Then, their biological properties were investigated using the standard micro dilution method, which determines the MIC. The conducted studies revealed that the MIC established for *E. coli* was equal to 6.25, 13.02, and 11.79 mg L^−1^ with respect to AgNPs with an average size of 7, 29, and 89 nm, respectively. In turn, the MIC detected for *S. aureus* increased with the AgNP size from 7.5 to 33.71 mg L^−1^. It is worth mentioning that these studies also revealed that the efficiency of AgNPs obtained with the use of GA was lower towards *S. aureus* than towards *E. coli*. The results of our studies reveal that larger AgNPs can be more efficient in the deactivation of bacteria and pathogenic fungi than smaller AgNPs with the same surface coating and charge.

Generally, scientific works showed that smaller AgNPs are more toxic because they are prone to oxidative dissolution and generate more silver ions, which in turn are considered to be a real toxic factor [72,73]. Moreover, Bae et al. [20], conducting studies on the impact of AgNPs on *E. coli* (ATCC8739), noticed that the amount of uptaken AgNPs with sizes lower than 14 nm was higher than for AgNPs with sizes of 90 and 140 nm. Morones et al. [70] indicated that AgNPs mainly in the size range of 1–10 nm attach to the surface of the cell membrane of Gram-negative bacteria and drastically disturb their proper functioning, such as permeability and respiration. Then, AgNPs penetrate inside bacteria and cause damage possibly by interacting with sulfur- and phosphorous-containing compounds such as DNA [70]. On the other hand, de Lima et al. [74] pointed out that there are some exceptions to this trend and it was documented that some smaller particles might be used more safely than larger ones [75]. In turn, Perni et al. [54] noticed that three types of cysteine-capped AgNPs of diverse size distribution and an average size (ca. 5 nm and 15 nm) gave the same value of MIC and MBC in experiments carried out on *E. coli* and *S. aureus*. Karlsson et al. [75] emphasized that it must be considered that factors other than size could be involved in the modulation of nanoparticle toxicity.

Hence, in the next stage of our studies, the impact of the surface charge of AgNPs on their biocidal activity towards the investigated pathogens was considered. CHSB1AgNPs, CHSB2AgNPs, LIZSBAgNPs, and ARGSBAgNPs were positively charged. CYSSBAgNPs were characterized by a positive surface charge only under acidic conditions, whereas at pH 7.4 they were negatively charged [27]. The rest of the investigated nanoparticles exhibited a negative surface charge independently of their surface coating (Table 2). Comparing the AgNPs of larger average sizes, one can notice that the negatively charged TCAgNPs were more biocidal for *E. coli* than the positively charged CHSB1AgNPs. This dependence was not maintained for *S. aureus.* In turn, the MIC attained the values of 45 and 50 mg L^−1^ (Table 3), which indicates that the effectiveness of TCAgNPs and CHSB1AgNPs was comparable. In turn, analyzing the results obtained for the AgNPs of the same surface properties but a smaller average size, one can see that the negatively charged TCSBAgNPs are more biocidal for *E. coli* than the positively charged CHSB2AgNPs (Table 3). This result is consistent with the data obtained for the larger AgNPs of the same surface properties. Nevertheless, the positively charged CHSB2AgNPs seem to be less active towards *S. aureus* than the negatively charged TCSBAgNPs, whereas in the case of *C. albicans* the relationship is the opposite and CHSB2AgNPs are more fungicidal than TCSBAgNPs.

Taking into consideration other oppositely charged AgNPs of diverse surface coating, one can notice that the positively charged LYZSBAgNPs are the most biocidal for *E. coli* among all investigated AgNPs (Table 3). ARGSBAgNPs also exhibit strong biocidal activity towards *E. coli*, which was determined based on the values of MIC and MBC. Nevertheless, the MIC and MBC values determined after *E. coli* exposure to ARGSBAgNPs are higher than those determined for the negatively charged TAAgNPs. Thus, one can conclude that the negatively charged, 12-nm-sized TAAgNPs are more biocidal for *E. coli* than the positively charged, 13-nm-sized ARGSBAgNPs.

*S. aureus* turned out to be the most sensitive to exposure to ARGSBAgNPs because the values of MIC and MBC were the lowest among all those established for each type of AgNP (Table 3). It is worth mentioning that the MIC values determined for TAAgNPs and CFGAAgNPs were significantly lower than the MIC of ARGSBAgNPs for *S. aureus* (Table 3). Nevertheless, the MBC values determined for the negatively charged AgNPs were almost two-fold higher than in the case of ARGSBAgNPs (Table 3). Surprisingly, the positively charged ARGSBAgNPs were not the most biocidal for *C. albicans*, which could be related to the completely different structure of the fungal cell wall and membrane compared with bacteria. The cell wall of Gram-positive bacteria is composed of a thick layer of peptidoglycan [76], while the cell wall of Gram-negative bacteria has a thin layer of peptidoglycan and an outer lipopolysaccharide membrane [76]. The fungal cell wall of the genus *Candida* is composed of 90% polysaccharides, mainly in the form of branched glucose polymers and unbranched glucosamine polymers and mannose polymers. The remaining 10% are proteins and lipids [77]. The negatively charged EGCGAgNPs exhibited the highest effectiveness towards deactivation of *C. albicans* because the lowest values of MIC and MBC were found for them.

Based on these findings, one can conclude that not all positively charged AgNPs are more biocidal towards the investigated pathogens than negatively charged AgNPs. It is also difficult to indicate that any pathogen is more sensitive to exposure to positively charged AgNPs. In the literature, it is speculated that the adhesion of AgNPs can be significantly promoted by the electrostatic attraction between negatively charged cell membranes of microorganisms and AgNPs with a positive surface charge, which provides positively charged AgNPs with stronger antibacterial activities compared with negatively charged AgNPs [16].

It was documented that positively charged AgNPs, regardless of their size, are more biocidal for prokaryotic cells [78] and eukaryotic cells [79,80]. This dependence of the surface charge of AgNPs on their toxicity was also observed in research conducted on mouse macrophage (RAW-264.7) and lung epithelial (C-10) cell lines [81]. However, an opposite effect was observed for histiocytic lymphoma (U-937) and human promyelocytic cells (HL-60) treated with diverse types of AgNPs of the same size distribution [53]. In the case of these studies, it was found that positively charged, cysteamine-stabilized AgNPs were less toxic for the cancer cells than two types of negatively charged AgNPs obtained with the use of a mixture of SB and TC. These results remain in good agreement with the findings obtained for *E. coli* and *S. aureus* exposed to CHSB2AgNPs and TCSBAgNPs.

It seems especially important to consider AgNPs’ biocidal activity towards prokaryotic and eukaryotic cells taking into account the chemistry and biological activity of their stabilizing agents. The information about the biological activity of chemicals used for the preparation of the investigated AgNPs is presented in Table 4. Several AgNPs were prepared using SB, which is a toxic substance. Each type of AgNP obtained using SB (Table 1) was stabilized by other chemicals. The efficiency of stabilization was confirmed by the electrokinetic characteristic involved in the determination of the zeta potential (Table 2). Despite the fact that cysteamine (CH) and cysteine (CYS) possess well-documented toxicity (Table 4), the AgNPs stabilized by them were not the most active towards the investigated pathogens. The expected enhancement of biocidal properties arising from the connection of silver to the biologically active aminothiol and amino acid was not observed in this part of the study. One can hypothesize that, due to the reaction of the thiol moiety leading to the chemisorption of cysteamine and cysteine on the surfaces of AgNPs, the biological activity of these compounds was reduced. It is worth mentioning that the bactericidal properties of cysteine-stabilized AgNPs were also studied by Perni et al. [54]. The authors observed that, regardless of the method used to synthesize AgNPs, the values of MIC and MBC determined for a given bacterium were equal to each other and attained 43.2 mg L^−1^ and 21.6 mg L^−1^ for *E. coli* and *S. aureus*, respectively.

L-Lysine and L-arginine, as opposed to L-cysteine, are not toxic for prokaryotic cells [82,83,84,85]. Nevertheless, numerous derivatives of these basic amino acids exhibit antibacterial properties and amplify the toxicity of other biologically active compounds (Table 4). In our studies, LYZSBAgNPs and ARGSBAgNPs turned out to be the most biocidal for each type of tested pathogen, which was an unexpected finding with respect to the biological activity of the considered amino acids. Previously, Tanvir et al. [86] studied the antibacterial properties of morphologically different AgNPs stabilized by selected compounds, including poly-L-arginine. The authors reported that modification of citrate- and PVP-stabilized spherical and prismatic AgNPs by poly-L-arginine enhances their biocidal activity towards *E. coli*, *P. aeruginosa*, and *Salmonella enterica*. By comparing these reports with our outcomes, one can conclude that both L-arginine and poly-L-arginine amplify the bactericidal properties of AgNPs. In our case, the citrate-stabilized TCSBAgNP and TCAgNPs were also less biocidal than ARGSBAgNPs for *S. aureus* and *C. albicans* (Table 3).

**Table 4:** Examples of the biological activity of stabilizing agents applied for the preparation of AgNPs.

Name of Compound	Examples of Biological Activity of the Compound and Its Derivatives	Ref.
cysteamine hydrochloride	Improvement of the bactericidal efficacy of intra-canal medicaments against *E.faecalis* Induction of apoptosis in cells treated with CH at the level of 10^−4^–10^−3^ M	[87,88]
L-cysteine	Enhancement of bactericidal effects of hydrogen peroxide in *E. coli* Inhibition of mycelial growth of the pathogenic fungus affecting grapevines *Eutypa lata* Inhibition and eradication of *C.albicans* biofilms Amplification of antibiotics activity against the Gram-negative bacteria *Persisters*	[89,90,91,92,93]
L-lysine	Induction of the bactericidal activity of antimicrobial peptides containing repetitive lysine–tryptophan motifs Higher antibacterial efficacy of materials containing poly-l-lysine chains Antimicrobial activity against phytopathogenic Gram-negative bacteria, e.g., the Gram-negative phylum Proteobacteria detected for epsilon-poly-lysine Induction of synergistic antifungal effects by epsilon-poly-lysine combined with chitooligosaccharide and mesoporous silica	[94,95,96,97,98]
L-arginine	Induction of the bactericidal activity of antimicrobial peptides containing repetitive arginine–tryptophan motifs Bactericidal properties by nanolayered materials containing poly-L-arginine Enhancement of bactericidal properties of AgNPs by poly-L-arginine Biological activity towards *C. albicans* by arginine-enriched antimicrobial peptides	[86,99,100,101]
trisodium citrate	Inhibition of growth of Gram-negative and Gram-positive bacteria and pathogenic fungi, including *C. albicans*, by high-concentration solutions of trisodium citrate Local anticoagulation properties by binding Ca^2+^	[102]
ascorbic acid	Antibacterial properties towards diverse strains of bacteria Dose-dependent antioxidant and prooxidant properties Modulation of pathogenicity markers of *Candida albicans* Enhancement of antifungal and antioxidant properties of curcumin	[103,104,105,106]
gallic acid	Dose-dependent antioxidant and prooxidant properties Well-established antibacterial, antifungal, and anti-inflammatory properties Inhibition of carcinogenesis in animal models and in vitro cancerous cell lines	[106,107]
(−)-epicatechin-3-gallate	Antibacterial and antiviral properties Documented antifungal properties towards *Candida* isolates Antioxidant properties Induction of cancer cell apoptosis Neuroprotective properties arising from the inhibition of protein fibrillation processes	[108,109,110]
tannic acid	Inhibition of bacteria growth and suppression of the mutagenesis in *E. coli* Antifungal properties against *P. digitatum* Antioxidant properties and ability to scavenge free radicals Antiamylogenic activity and possibilities to destabilize abnormal protein fibrils	[111,112,113,114,115]
caffeine	Widely described antibacterial and antifungal properties Antiviral properties towards selected viruses Dose-dependent antioxidant and pro-oxidant properties	[116,117,118,119]
hydroxylamine mine hydrochloride	A strong mutagen with reported activity against phages, viruses, bacteria, fungi, protozoa, and plants	[120]
sodium hexametaphosphate	Inorganic permeabilizer increasing the permeability of biological membranes Enhancer of bactericidal properties of other biologically active substances	[121,122,123,124]

All the antioxidants applied during the synthesis of AgNPs possess well-established biocidal properties (Table 4). For this reason, it was expected that AgNPs prepared with their use should be more biocidal for the tested pathogens than TCSBAgNPs and TCAgNPs, which were coated with citrate anions. It was found that TCAAAgNPs were the least effective in the group of antioxidant-synthesized AgNPs. The MIC and MBC values found for these AgNPs were comparable to or higher than the values established for TCAgNPs despite their smaller size. This observation is obvious taking into account that the TCAAAgNPs were coated with citrate anions.

The efficacy of TAAgNPs against *E. coli* was comparable to that detected for ARGSBAgNPs. It is worth noting that the MIC and MBC of TAAgNPs determined in the case of *S. aureus* were equal to 5 and 100 mg L^−1^, respectively (Table 3). This indicates that TAAgNPs were less effective in the deactivation of Gram-positive than Gram-negative bacteria. On the other hand, the fungicidal properties of TAAgNPs and GAAgNPs were comparable and noticeably worse than the antifungal activity of EGCGAgNPs and CAFGAAgNPs (Table 3). These outcomes show that the biocidal properties of AgNPs towards specific pathogens can be tuned by the selection of proper antioxidants.

A stronger activity towards one type of pathogen was noticed in the case of HHAgNPs, which were synthesized using a carbon-free inorganic compound. Analyzing the results presented in Table 3, one can observe that, to inhibit the growth of *E. coli*, a noticeably lower concentration of HHAgNPs was needed than in the case of *S. aureus* and *C. albicans*. Interestingly the MBC value detected for HHAgNPs-treated *E. coli* was equal to 30 mg L^−1^ (Table 3). This value was ca. twofold higher than the one detected by Kujda et al. [52] for AgNPs obtained according to the same preparation protocol and characterized by a comparable size distribution. In our opinion, this discrepancy between obtained MBC values arises from the application of different assays and parameters (e.g., incubation time, pathogen concentration) for MBC determination. It is also worth mentioning that Kujda et al. [52] showed that the values of MBC determined for given AgNPs using an established assay are different for diverse strains of *E. coli* and usually higher for antibiotic-resistant strains. Furthermore, the authors showed that AgNPs obtained using sodium hexametaphosphate, which is a common permeabilizer (Table 4), exhibit improved biocidal activity towards the tetracycline-resistant strain of *E. coli*. In the study by Kujda et al. [52], AgNPs stabilized by sodium hexametaphosphate (SH) were the most biocidal for *E. coli* among all investigated negatively charged nanoparticles.

A sodium-hexametaphosphate-induced improvement in AgNP toxicity was also confirmed in further studies conducted by Mendes-Gouvêa et al. [124]. The authors documented that the composites of AgNPs and sodium hexametaphosphate significantly reduced the formation of biofilms of *C. albicans* and *Streptococcus mutans*. In this way, the enhancement of the biocidal activity of AgNPs by sodium hexametaphosphate was confirmed for Gram-positive bacteria and fungi.

SHSHAgNPs stabilized by sodium hexametaphosphate (SH) and investigated in these studies exhibited satisfactory biocidal properties (Table 3). Nevertheless, the MBC values determined for SHSHAgNPs in the experiments with *E. coli* were comparable to the results obtained for TCSBAgNPs considered as model nanoparticles (Table 3). In turn, positively charged ARGSBAgNPs gave noticeably smaller values of MBC than SHSHAgNPs in experiments with *S. aureus* and *C. albicans*. Considering the nontoxicity of ARG and sodium-hexametaphosphate-induced enhancement of membrane permeability, it can be speculated that these data indicate the dominant role that the surface charge of AgNPs plays in their biological activity. However, one can observe a lack of repeatability of this dependence in the case of a comparison of the MBC values for SHSHAgNPs and, e.g., positively charged CYSSBAgNPs.

The obtained results suggest that the biological effect induced by AgNPs strongly depends on their physicochemical properties as well as the morphology and physiology of the pathogens exposed to them.

Taking the obtained values of MIC and MBC into account, one can conclude that negatively charged TCAAAgNPs of medium size were the least biocidal for the investigated pathogens. Negatively charged EGCGAgNPs obtained with the use of common antioxidants exhibited the best fungicidal properties. In turn, positively charged ARGSBAgNPs were characterized by the highest bactericidal and fungicidal properties towards the investigated pathogens. Overall, one can also notice that Gram-negative *E. coli* was the most sensitive to exposure to AgNPs.

## 4. Conclusions

A chemical reduction of silver ions in the presence of diverse biologically active compounds allows for the production of AgNPs with the desired size, shape, and surface properties. AgNPs possess antibacterial and antifungal properties regardless of the method used to synthesize them. Nevertheless, the effectiveness of the deactivation of pathogens by AgNPs is correlated to their physicochemical properties. The conducted studies reveal that larger AgNPs can be more biocidal than smaller ones. It was also established that, in some cases, positively charged AgNPs are less toxic than negatively charged AgNPs with the same size distribution. Based on these results, it was concluded that the stabilizing agents play a dominant role in AgNP toxicity.

Each type of AgNP investigated in this research was obtained using biologically active compounds. It was observed that, in some cases, the expected enhancement of AgNP toxicity, arising from stabilization using compounds with well-documented biocidal properties, did not occur. It was established that the loss of biological activity of stabilizing agents may be related to their chemisorption on the AgNP surface or the oxidation that occurred during the nanoparticles’ synthesis.

It was found that the positively charged, arginine-stabilized AgNPs (ARGSBAgNPs) were the most toxic. The values of MIC and MBC determined for both types of bacteria and fungi treated by ARGSBAgNPs were comparable for all investigated systems. Therefore, it was concluded that ARGSBAgNPs exhibit universal activity towards the investigated pathogens. The strongest fungicidal properties were detected for the negatively charged EGCGAgNPs obtained using (−)-epigallocatechin gallate (EGCG). Some negatively charged AgNPs gave lower values of MIC and MBC than EGCGAgNPs in the treatment of Gram-positive and Gram-negative bacteria. Based on these facts, it was concluded that by applying a specific stabilizing agent one can tune the selectivity of AgNP toxicity towards the desired pathogen.

Generally, it was established that *E. coli* was more sensitive to exposure to AgNPs than *S. aureus* regardless of AgNP size and surface properties.

## Figures and Tables

**Table 2:** Types of silver nanoparticles (AgNPs) obtained using selected reagents and described reaction conditions.

Symbol	λ_max_	*d* (nm)	PdI	*D* (×10^−7^ cm^2^ s^−1^)	*d*_H_ (nm)	*μ*_e_ (μm cm (Vs))	ζ (mV)
CHSB1AgNPs	447	55 ± 9	0.17	1.29	50 ± 5	4.55 ± 0.12	70 ± 2
CHSB2AgNPs	403	12 ± 4	0.33	5.37	12 ± 1	3.21 ± 0.17	51 ± 2
CYSSBAgNPs	396	12 ± 3	0.25	5.85	11 ± 2	2.54 ± 0.28	40 ± 4
LYZSBAgNPs	402	16 ± 5	0.31	4.61	14 ± 3	1.26 ± 0.09	25 ± 2
ARGSBAgNPs	403	13 ± 5	0.38	4.96	13 ± 3	1.61 ± 0.06	31 ± 2
TCSBAgNPs	392	13 ± 5	0.38	5.85	11 ± 3	−2.78 ± 0.14	−45 ± 3
TCAgNPs	438	57 ± 10	0.18	1.22	53 ± 4	−3.03 ± 0.11	−47 ± 2
TCAAAgNPs	400	12 ± 4	0.33	6.44	10 ± 2	−2.53 ± 0.03	−40 ± 1
GAAgNPs	397	12 ± 4	0.33	6.44	10 ± 3	3.29 ± 0.02	−52 ± 2
EGCGAgNPs	405	15 ± 4	0.27	4.29	15 ± 2	−3.87 ± 0.06	−61 ± 1
TAAgNPs	412	13 ± 5	0.39	5.37	12 ± 1	−3.30 ± 0.23	−52 ± 3
CFGAAgNPs	400	17 ± 4	0.24	4.29	15 ± 1	−3.16 ± 0.09	−49 ± 2
GLAgNPs	429	23 ± 8	0.35	2.93	22 ± 2	−3.17 ± 0.05	−50 ± 1
HHAgNPs	403	13 ± 3	0.23	5.85	11 ± 1	−3.65 ± 0.09	−55 ± 2
SHSHAgNPs	398	11 ± 3	0.27	6.44	10 ± 2	−3.76 ± 0.12	−57 ± 4

Blue—AgNPs obtained using sodium borohydride (SB); yellow/orange—AgNPs obtained using trisodium citrate (TC); green—AgNPs obtained using selected antioxidants; grey—AgNPs obtained using glucose; red—AgNPs obtained using selected inorganic compounds.

**Table 3:** Biocidal properties of AgNPs expressed as the AgNP concentration established as minimum inhibitory concentration (MIC) and minimum bactericidal concentration (MBC) for a given type of AgNP and determined for *E. coli* (ATCC 25922), *S. aureus* (ATCC 292113), and *C. albicans* (ATCC 10231).

Symbol	*d* (nm)	*Escherichia coli*		*Staphylococcus aureus*		*Candida albicans*	
		MIC	MBC	MIC	MBC	MIC	MBC
CHSB1AgNPs	55 ± 9	25	45	45	45	45	45
CHSB2AgNPs	12 ± 4	35	75	45	100	20	65
CYSSBAgNPs	12 ± 3	45	100	80	100	100	100
LYZSBAgNPs	16 ± 5	5	10	5	100	10	60
ARGSBAgNPs	13 ± 5	20	30	25	30	25	30
TCSBAgNPs	13 ± 5	40	45	50	80	100	100
TCAgNPs	57 ± 10	15	35	100	100	50	50
TCAAAgNPs	12 ± 4	25	90	75	100	100	100
GAAgNPs	12 ± 4	40	100	40	100	100	100
EGCGAgNPs	15 ± 4	15	40	30	70	10	10
TAAgNPs	13 ± 5	5	15	5	100	80	100
CFGAAgNPs	17 ± 4	10	60	10	85	30	50
GLAgNPs	23 ± 8	25	60	50	100	10	100
HHAgNPs	13 ± 3	25	25	100	100	100	100
SHSHAgNPs	11 ± 3	5	40	15	55	35	100

Blue—AgNPs obtained using sodium borohydride (SB); yellow/orange—AgNPs obtained using trisodium citrate (TC); green—AgNPs obtained using selected antioxidants; grey—AgNPs obtained using glucose; red—AgNPs obtained using selected inorganic compounds.

## Data Availability

Data is contained within Supplementary material or are available on request from the corresponding author.

## Supplementary Materials

The following are available online at https://www.mdpi.com/article/10.3390/biom11101481/s1, 1. Synthesis of silver nanoparticles, Figure S1: Typical TEM micrographs and size distributions obtained based on microscopic analysis of: (a) CHSB1AgNPs, (b) CHSB2AgNPs, (c) CYSSBAgNPs, (d) LIZSBAgNPs, (e) ARGSBAgNPs, (f) TCSBAgNPs, (g) TCAgNPs, (h) TCAAAgNPs, (i) GAAgNPs, (j) EGCGAgNPs, (k) TAAgNPs, (l) CAFGAAgNPs, (m) GLAgNPs, (n) HHAgNPs, and (o) SHSHAgNPs.
