# Supplementary material for: Antibacterial and Antifungal Properties of Silver Nanoparticles—Effect of a Surface-Stabilizing Agent
Source: Biomolecules. 2021 Oct 7;11(10):1481. doi: 10.3390/biom11101481 (PMC8533414; doi:10.3390/biom11101481)
Supplement: Supplementary file 1 [file biomolecules-11-01481-s001.zip › biomolecules-1392755-supplementary.pdf]

Supplementary Materials

Antibacterial and Antifungal Properties of Silver Nanoparticles—Effect of a Surface-Stabilizing Agent

Agnieszka Gibała [1,2,*], Paulina Żeliszewska [2], Tomasz Gosiewski [1], Agnieszka Krawczyk [1], Dorota Duraczyńska [2], Joanna Szaleniec [3], Maciej Szaleniec [2] and Magdalena Oćwieja [2]

[1] Department of Molecular Medical Microbiology, Chair of Microbiology, Faculty of Medicine, Jagiellonian University Medical College, Czysta 18, 31-12 Krakow, Poland; tomasz.gosiewski@uj.edu.pl (T.G.); agnieszka.krawczyk@doctoral.uj.edu.pl (A.K.)

[2] Jerzy Haber Institute of Catalysis and Surface Chemistry, Polish Academy of Sciences, Niezapominajek 8, 30-239 Krakow, Poland; paulina.zeliszewska@ikifp.edu.pl (P.Ż.); dorota.duraczynska@ikifp.edu.pl (D.D.); maciej.szaleniec@ikifp.edu.pl (M.S.); magdalena.ocwieja@ikifp.edu.pl (M.O.)

[3] Department of Otolaryngology, Faculty of Medicine, Jagiellonian University Medical College, Jakubowskiego 2, 30-688 Krakow, Poland; joanna.szaleniec@uj.edu.pl

* Correspondence: agnieszka.gibala@doctoral.uj.edu.pl; Tel.: +48-509681183

1. Synthesis of Silver Nanoparticles

Cysteamine-stabilized silver nanoparticles (CHSB1AgNPs) were synthesized using sodium borohydride (SB) as a reducing agent and cysteamine hydrochloride (CH) as a stabilizing agent [53]. For this purpose, 2.5 mL of 5 mM freshly prepared aqueous solution of SB was added dropwise to 200 mL of 2.5 mM aqueous solution of silver nitrate. After, 10 min of vigorous stirring at room temperature, 10 mL of 1.5 mM of freshly prepared aqueous solution of CH was added dropwise to the newly formed AgNPs. The mixing was continued for additional 60 min.

Cysteamine-stabilized silver nanoparticles (CHSB2AgNPs) were prepared according to the procedure described above with some modifications. Briefly, 2.5 mL of 10 mM solution of SB prepared in MilliQ water of temperature equal to 4 °C was introduced immediately to 160 mL of 2.5 mM solution of silver nitrate. Then, 5 mL of 1.42 mM aqueous solution of CH was introduced to the reaction mixture. The stirring was continued for next 60 min.

Cysteine-stabilized silver nanoparticles (CYSBAgNPs) were prepared under anaerobic conditions and at ambient temperature [27]. For this reason, silver nitrate, SB and cysteine (CY) solutions were prepare using MilliQ water which was purged with argon. Maintaining anaerobic conditions, 12 mL of 10 mM SB was introduced into 160 mL of silver nitrate solution of concentration 2.8 mM which was stirred vigorously. Then, 6 mL of 1.45 mM cysteine solution was added to the silver nanoparticle suspension. The stirring under anaerobic conditions was carried out for 70 min.

Lysine-stabilized silver nanoparticles (LYZSBAgNPs) were synthesized under ambient conditions using SB as a reducing agent and L-lysine (LYZ) as a stabilizing agent. At the beginning, 200 mL of 2.5 mM silver nitrate solution was mixed with 8 mL of 1.6 mM solution of LYZ. Then, 12 mL of 9.5 mM SB was added dropwise into the reaction mixture maintaining a dynamic stirring. It was established that the formation of LYZSBAgNPs was finished after 40 min of stirring.

Arginine-stabilized silver nanoparticles (ARGSBAgNPs) were synthesized under ambient conditions using SB as a reducing agent and L-arginine (ARG) as a stabilizing agent. For this purpose, 200 mL of 2.5 mM silver nitrate solution was mixed with 5 mL of 1.2 mM solution of ARG. Then, 15 mL of 7.5 mM SB was added dropwise into the reaction mixture maintaining a dynamic stirring. The mixing was continued for additional 40 min.

Citrate-stabilized silver nanoparticles (TCSBAgNPs) were prepared according to the Lee and Meisel method [41] using SB as a reducing agent and trisodium citrate (TC) as a stabilizing agent. Briefly, 200 mL of 8 mM freshly prepared SB aqueous solution was mixed with 200 mL of 20 mM TC solution. Then, while stirring, the 100 mL of 1 mM silver nitrate solution was added dropwise into the reduction mixture for 20 min. The reaction was allowed to proceed, while being continually stirred, under ambient conditions for 90 min.

Citrate-stabilized silver nanoparticles (TCAgNPs) were obtained according to the Turkevich method developed by some modifications [42]. Briefly, 90 mL of 1.18 mM silver nitrate solution was heated to 100 °C under a reflux condenser. Then, while stirring 4 mL of 29 mM TC solution was added rapidly to the boiled silver nitrate solution. The heating under stirring was continued for 60 min and then obtained suspension was cooled down to the room temperature in an ice bath.

Citrate-stabilized silver nanoparticles (TCAAAgNPs) were obtained using L (+)-ascorbic acid (AA) as a reducing agent and TC as a stabilizing agent. For this purpose, 1 mM solution of silver nitrate, 3 mM of AA and 1 wt % of TC were prepared using MilliQ water water. Then,1 mL of AA solution was added dropwise to the mixture containing 80 mL of silver nitrate solution, 20 μL of TC and 20 μL of 25 wt % ammonia solution placed on amagnetic stirrer. Stirring was continued for another 30 min when the color of the mixture changed to yellowish-gray.

Gallic acid-stabilized silver nanoparticles (GAAgNPs) were prepared using gallic acid (GA) which plays a dual role of reducing and stabilizing agent of formed nanoparticles [55]. Thus, 10 mL of 1 mM aqueous solution of GA was

mixed with 80 mL of 1 mM aqueous solutions of silver nitrate at room temperature. Then, 40 μL of 25 wt % ammonia solution was introduced to the mixture while stirring. The stirring was continued for another 40 minutes.

(−)-epigallocatechin gallate-stabilized silver nanoparticles (EGCGAgNPs) were prepared in a similar manner as GAAgNPs. 10 mL of 1mM aqueous solution of EGCG was mixed with 150 mL of 1 mM aqueous solutions of silver nitrate at room temperature. Then, 30 μL of 25 wt % ammonia solution was introduced to the mixture while stirring. The stirring was continued for another 40 min.

Tannic acid-stabilized silver nanoparticles (TAAgNPs) were prepared using tannic acid (TA) which plays a dual role of a reducing and stabilizing agent of formed nanoparticles [49]. In a nutshell, 40 mL of 0.5 mM aqueous solution of TA was introduced to 320 mL of 11 mM silver nitrate solution. After, the dynamic stirring 30 μL of 25 wt % ammonia solution was introduced to the obtained reaction mixture. The stirring was continued for another 30 min.

Caffeine-stabilized silver nanoparticles (CFGAAgNPs) were prepared using GA as a reducing agent and caffeine (CF) as a stabilizing agent. For this purpose, 10 mL of 1 mM GA solution was mixed with 10 mL of 2.3 mM CF solution. Then, the reducing mixture was added to 150 mL of 1 mM silver nitrate solution. After, the dynamic stirring 40 μL of 25 wt % ammonia solution was introduced to the obtained reaction mixture. The stirring was maintained over further 30 min.

Glucose-stabilized silver nanoparticles (GLAgNPs) were obtained according to the modified preparation method developed by Panáček et al. [57]. Thus, 20 mL of 5 mM aqueous solution of D-(+)-glucose (GL) was mixed with 200 mL of 1.2 mM aqueous solutions of silver nitrate at room temperature. Then, 20 μL of 25 wt % ammonia solution was introduced to the mixture while stirring. The stirring was continued for a further 50 min.

Hydroxylamine hydrochloride-stabilized silver nanoparticles (HHAgNPs) were synthesized according to the modified Leopold and Lendl [51] method using hydroxylamine hydrochloride (HH) as a reducing and stabilizing agent of nanoparticles. Briefly, 180 mL of 2 mM aqueous solution of HH was mixed with 500 μL of 1 mM sodium hydroxide. After, 2 min of the mixing, 15 mL of 12 mM silver nitrate solution was rapidly added to the reaction mixture. The stirring was continued for further 40 min.

Sodium hexametaphosphate-stabilized silver nanoparticles (SHSHAgNPs) were prepared according to the patented procedure [58] described further in details by Barbasz and Oćwieja [33]. The formation of AgNPs is conducted at elevated temperature and under acidic conditions using sodium hypophosphite (SH) as a reducing agent and sodium hexametaphosphate (SH) as a stabilizing agent. For this purpose, 20 mL of aqueous solution of 0.4 M of sodium hypophosphite was mixed with 300 mL of 3 mM solution of sodium hexametaphosphate. Then, the pH of the reaction mixture was decreased to 2.9 using sulfuric acid. Afterwards, the solution was heated to 40 °C while stirring and 100 mL of 10 mM silver nitrate was added to the mixture. The reaction mixture was maintained under heating for further 90 min.

References

27 Oćwieja, M.; Morga, M. Electrokinetic properties of cysteine-stabilized silver nanoparticles dispersed in suspensions and deposited on solid surfaces in the form of monolayers. *Electrochim. Acta* **2018**, *297*, 1000–1010, doi:10.1016/j.electacta.2018.11.213.

33 Oćwieja, M.; Barbasz, A. Sodium hexametaphosphate–induced enhancement of silver nanoparticle toxicity towards leukemia cells. *J. Nanopart. Res.* **2020**, *22*, 167, doi:10.1007/s11051-020-04903-w.

41 Lee, P.C.; Meisel, D. Adsorption and surface-enhanced Raman of dyes on silver and gold sols. *J. Phys. Chem.* **1982**, *86*, 3391–3395, doi:10.1021/j100214a025.

42 Turkevich, J.; Stevenson, P.C.; Hillier, J. A study of the nucleation and growth processes in the synthesis of colloidalgold. *Discuss. Faraday Soc.* **1951**, *11*, 55–75, doi:10.1039/df9511100055.

49 Dadosh, T. Synthesis of uniform silver nanoparticles with a controllable size. *Mater. Lett.* **2009**, *63*, 2236–2238, doi:10.1016/j.matlet.2009.07.042.

51 Leopold, A.N.; Lendl, B.A. New method for fast preparation of highly surface-enhanced Raman scattering(SERS) active silver colloids at room temperature by reduction of silver nitrate with hydroxyl amine hydrochloride. *J. Phys. Chem. B* **2003**, *107*, 5723–5727, doi:10.1021/jp027460u.

53 Barbasz, A.; Oćwieja, M.; Roman, M. Toxicity of silver nanoparticles towards tumoral human cell lines U-937 and HL-60. *Colloids Surf. B Biointerfaces* **2017**, *156*, 397–404, doi:10.1016/j.colsurfb.2017.05.027.

55 Li, D.; Liu, Z.; Yuan, Y.; Liu, Y.; Niu, F. Green synthesis of gallic acid-coated silver nanoparticles with high antimicrobial activity and low cytotoxicity to normal cells. *Process. Biochem.* **2015**, *50*, 357–366, doi:10.1016/j.procbio.2015.01.002.

57 Panacek, A.; Kvitek, L.; Prucek, R.; Kolář, M.; Večeřová, R.; Pizfuirova, N.; Sharma, V.K.; Nevecna, T.; Zboril, R. Silver colloid nanoparticles: Synthesis, characterization, and their antibacterial activity. *J. Phys. Chem. B* **2006**, *110*, 16248–16253, doi:10.1021/jp063826h.

58 Adamczyk, Z.; Kujda. M.; Oćwieja. M. Sposób wytwarzania stabilnych suspensji nanocząstek srebra oraz zastosowanie stabilnych suspensji nanocząstek srebra do celów biobójczych. Polish Patent PL 224713, 15 June 2016.

Figure S1. Typical TEM micrographs and size distributions obtained based on microscopic analysis presenting: (**a**) CHSBAgNPs1, (**b**) CHSBAgNPs2, (**c**) CYSBAgNPs, (**d**) LIZSBAgNPs, (**e**) ARGSBAgNPs, (**f**) TCSBAgNPs, (**g**) TCAgNPs, (**h**) TCAAAgNPs, (**i**) GAAgNPs**,** (**j**) EGCGAgNPs, (**k**) TAAgNPs, (**l**) CAFGAAgNPs, (**m**) GLAgNPs, (**n**) HHAgNPs, (**o**) SHSHAgNPs.

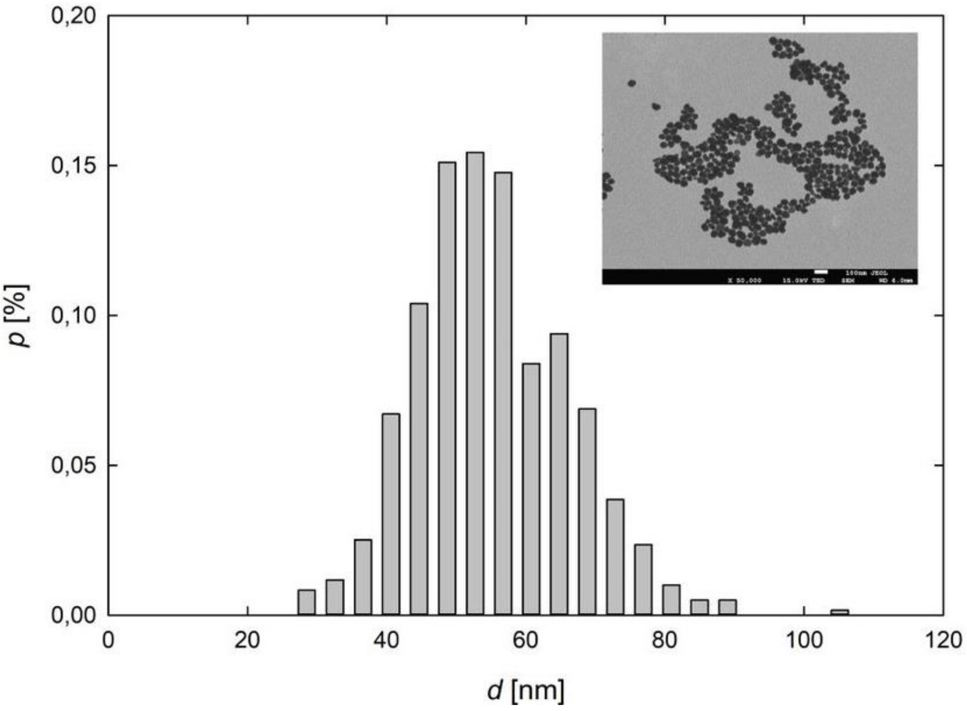

(**a**)

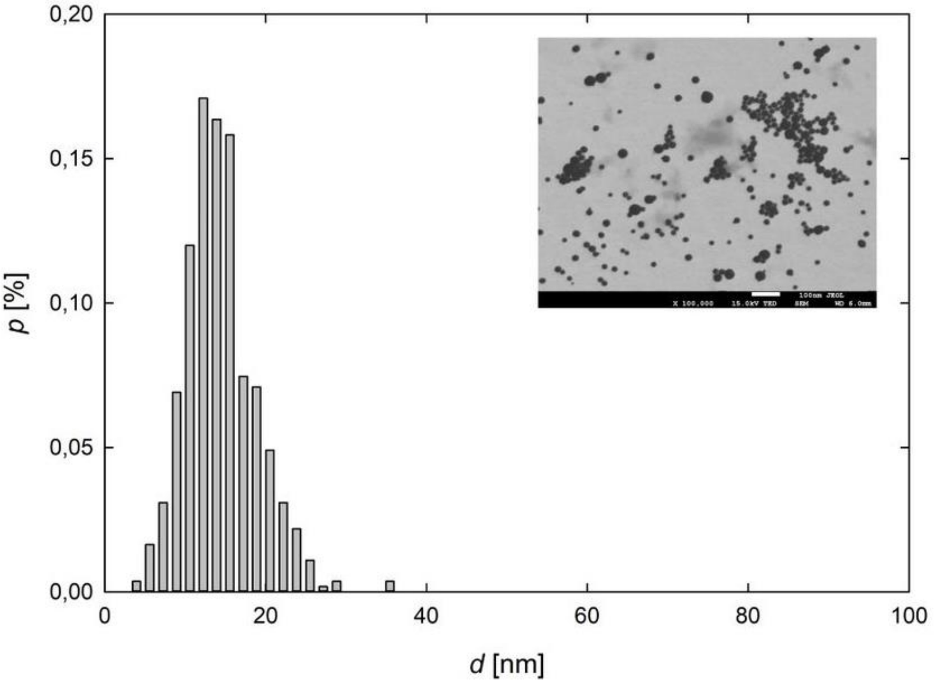
0,20
0,15
0,10
0,05
0,00
p [%]
0
20
40
60
80
100
d [nm]

(**b**)

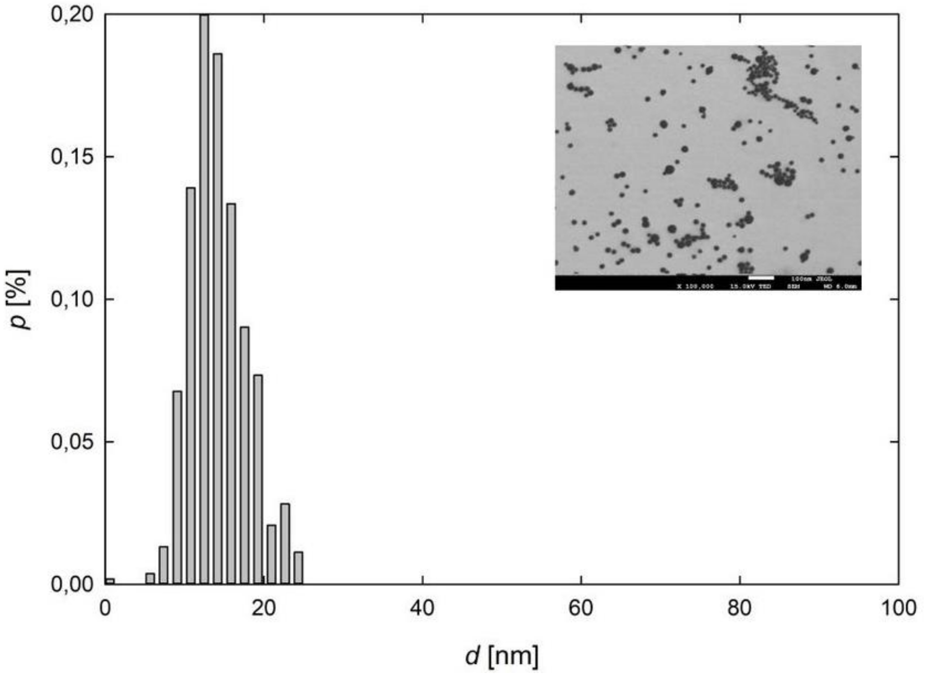
0,20
0,15
0,10
0,05
0,00
p [%]
0
20
40
60
80
100
d [nm]

(**c**)

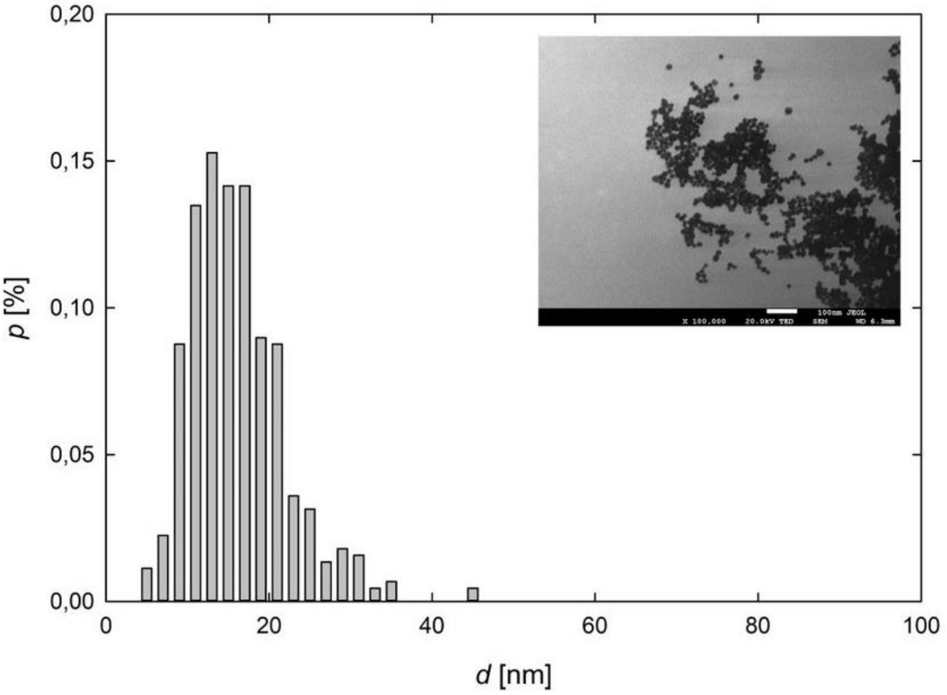
0,20
0,15
0,10
0,05
0,00
p [%]
0
20
40
60
80
100
d [nm]

(**d**)

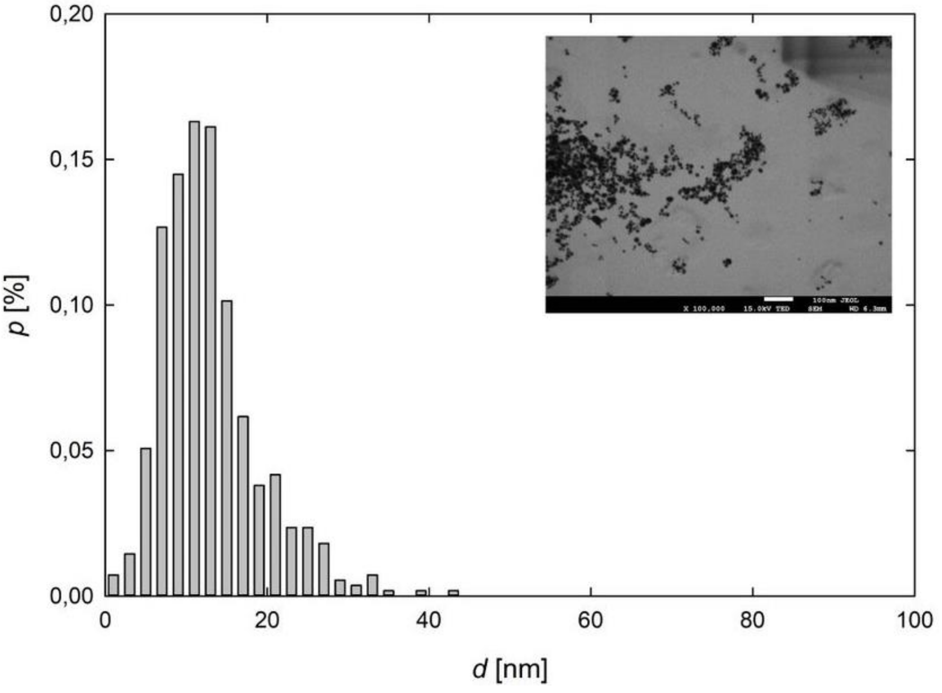
0,20
0,15
0,10
0,05
0,00
p [%]
0
20
40
60
80
100
d [nm]

(**e**)

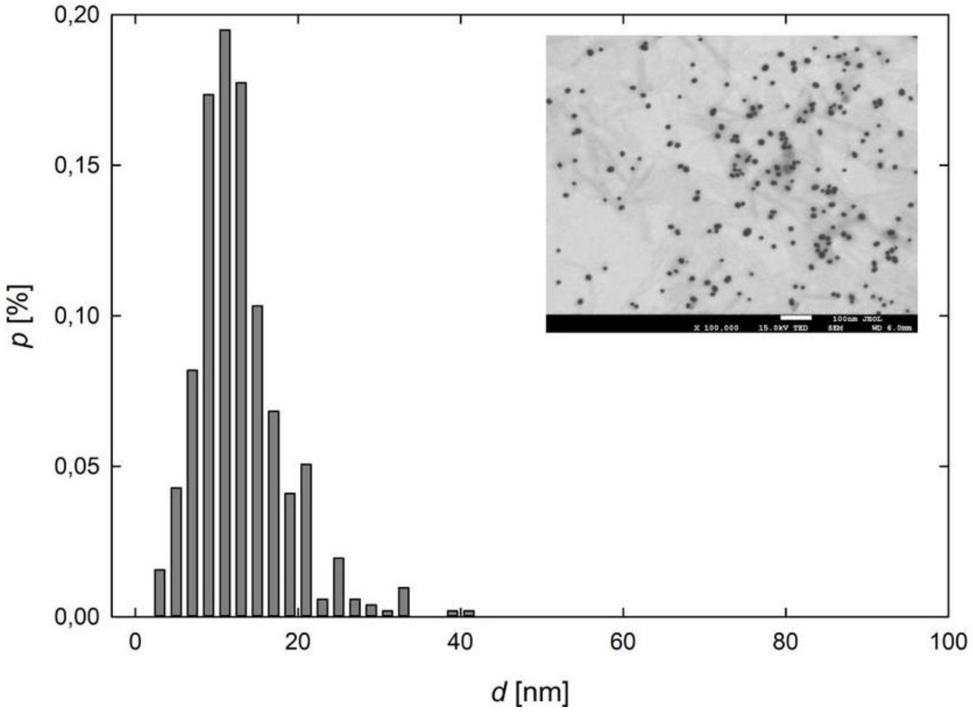
0,20
0,15
0,10
0,05
0,00
p [%]
0
20
40
60
80
100
d [nm]

(**f**)

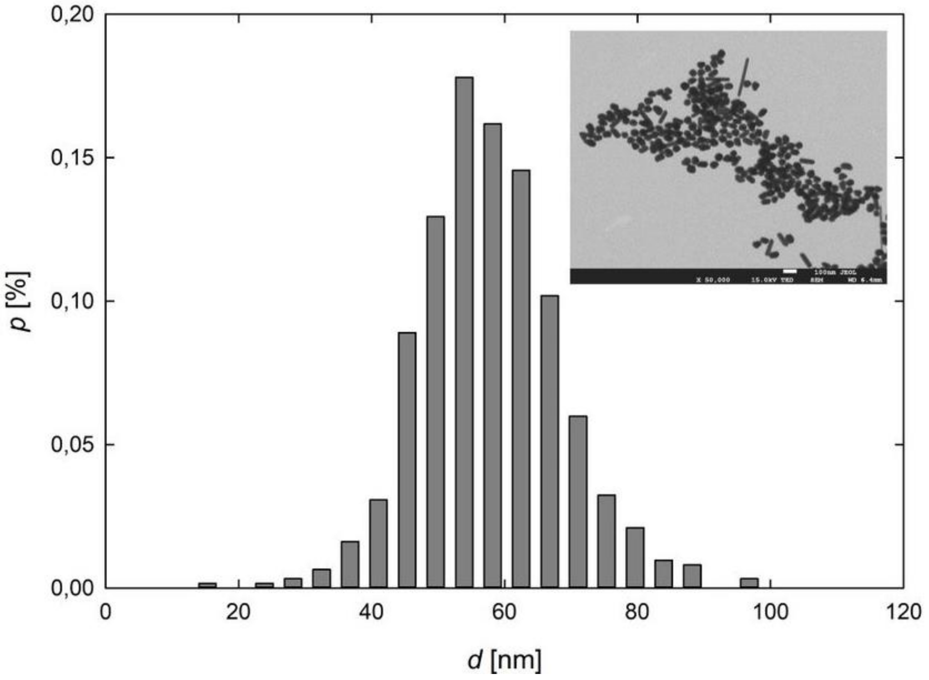
0,20
0,15
0,10
0,05
0,00
p [%]
0
20
40
60
80
100
120
d [nm]

(**g**)

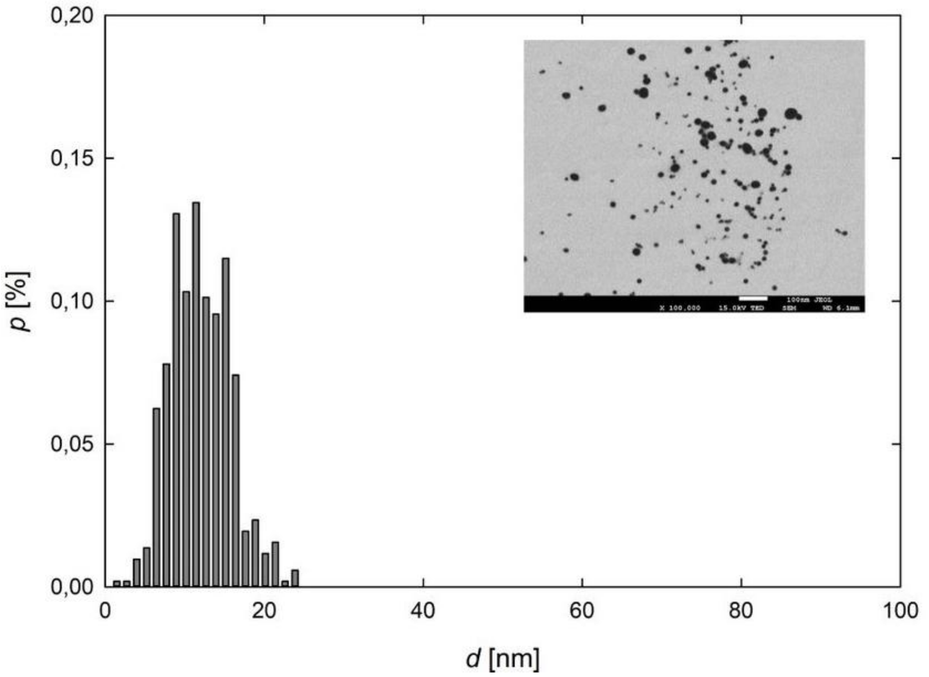
0,20
0,15
0,10
0,05
0,00
p [%]
0
20
40
60
80
100
d [nm]

(**h**)

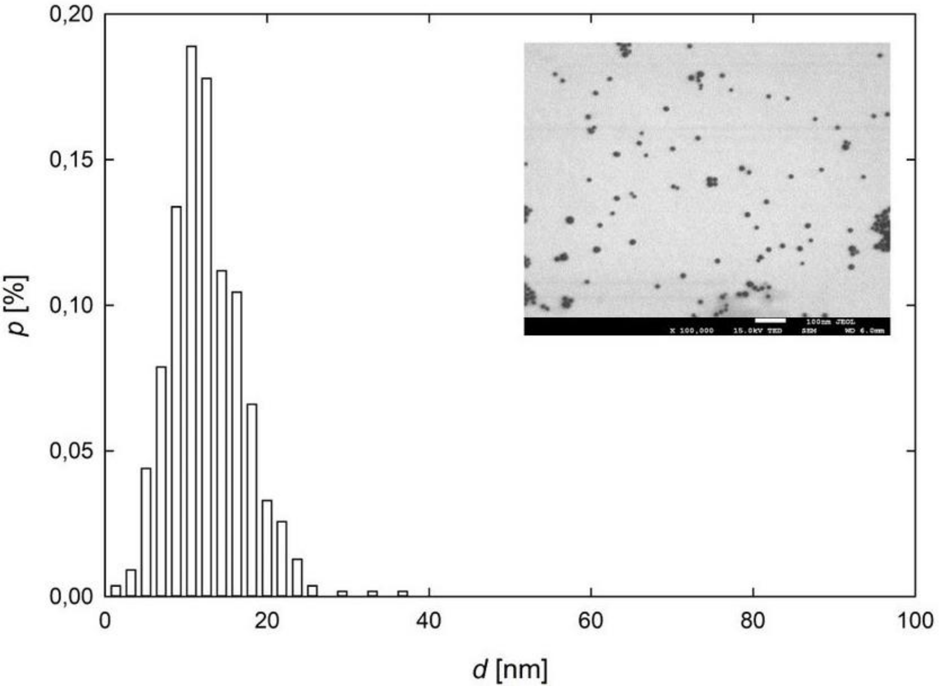
0,20
0,15
0,10
0,05
0,00
p [%]
0
20
40
60
80
100
d [nm]

(**i**)

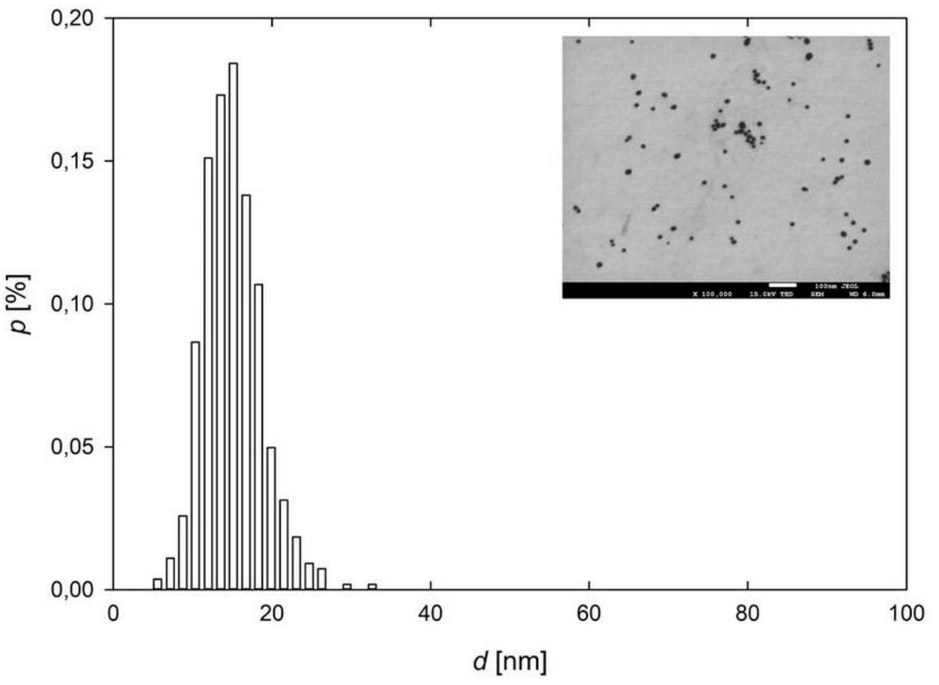

0,20
0,15
0,10
0,05
0,00
p [%]
0
20
40
60
80
100
d [nm]

(**j**)

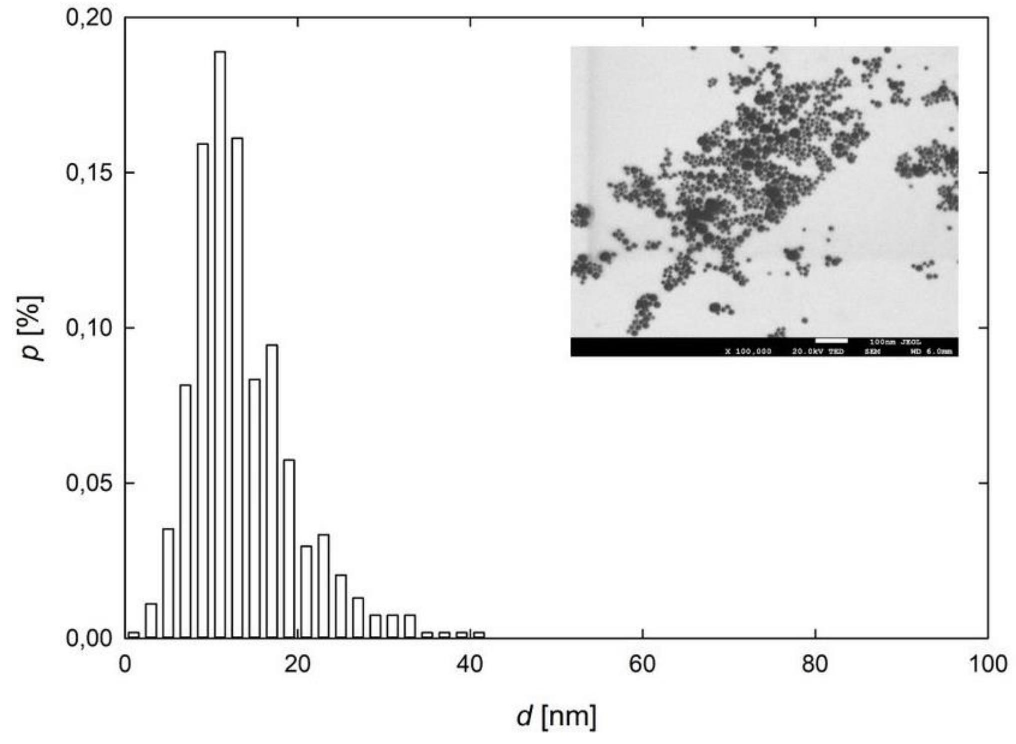

0,20
0,15
0,10
0,05
0,00
p [%]
0
20
40
60
80
100
d [nm]

(**k**)

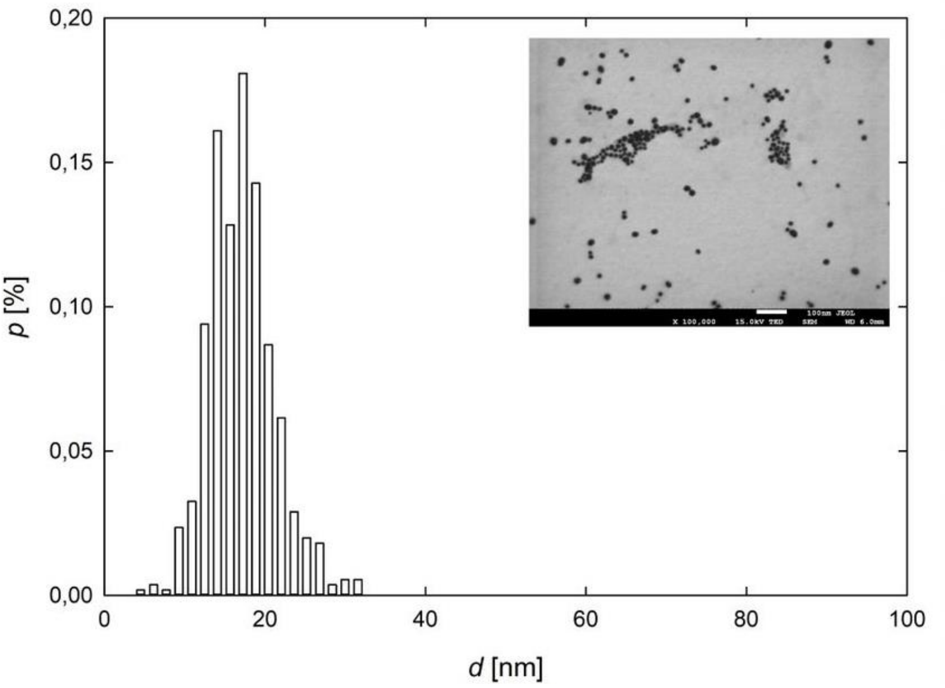
0,20
0,15
0,10
0,05
0,00
p [%]
0
20
40
60
80
100
d [nm]

(**l**)

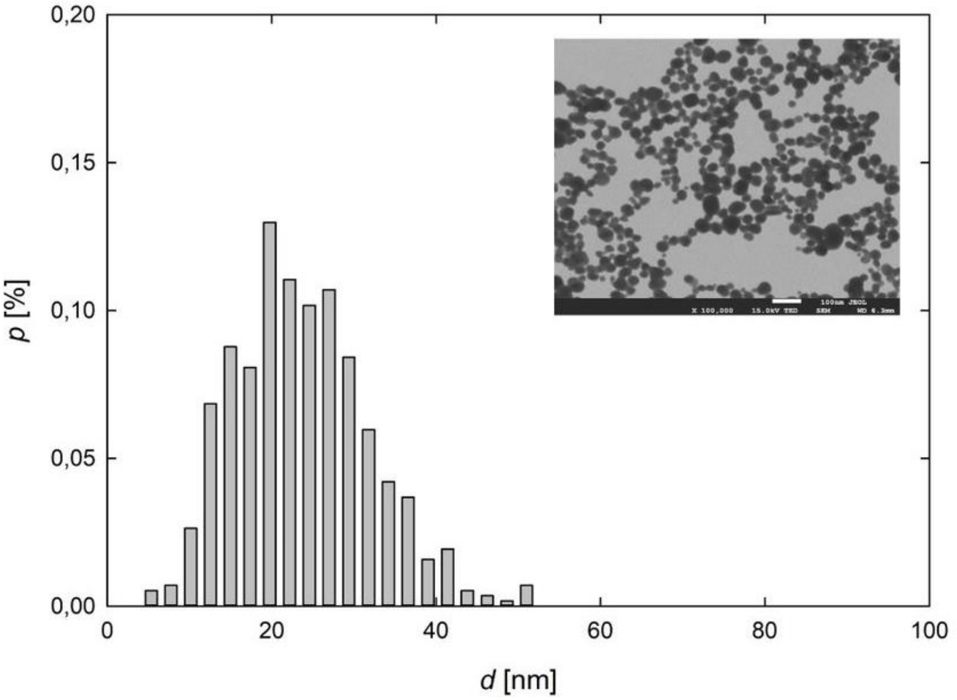
0,20
0,15
0,10
0,05
0,00
p [%]
0
20
40
60
80
100
d [nm]

(**m**)

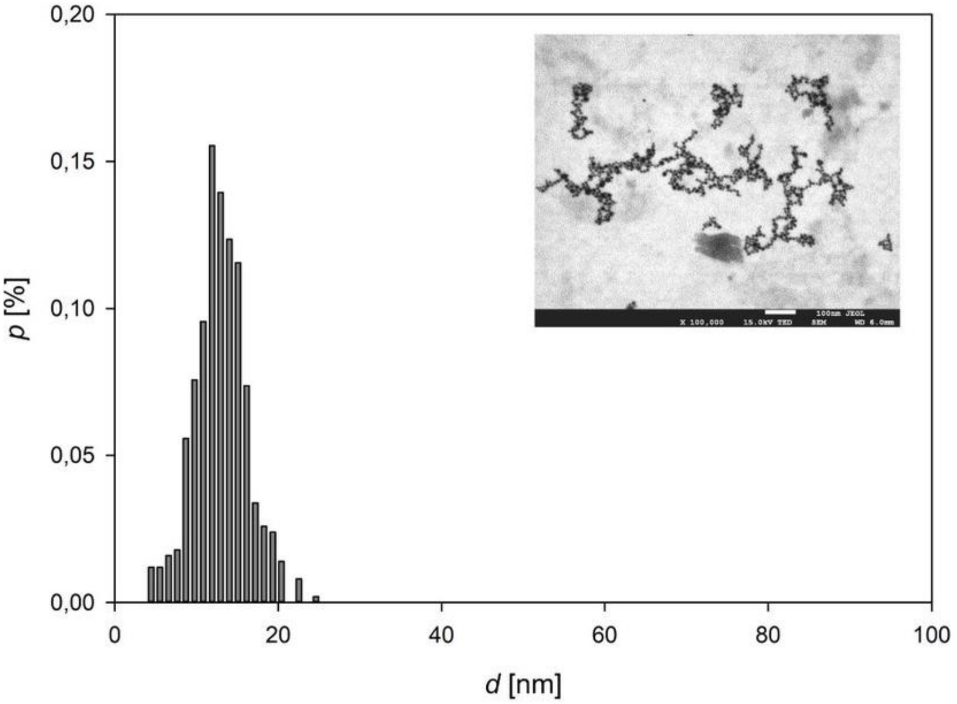
0,20
0,15
0,10
0,05
0,00
p [%]
0
20
40
60
80
100
d [nm]

(**n**)

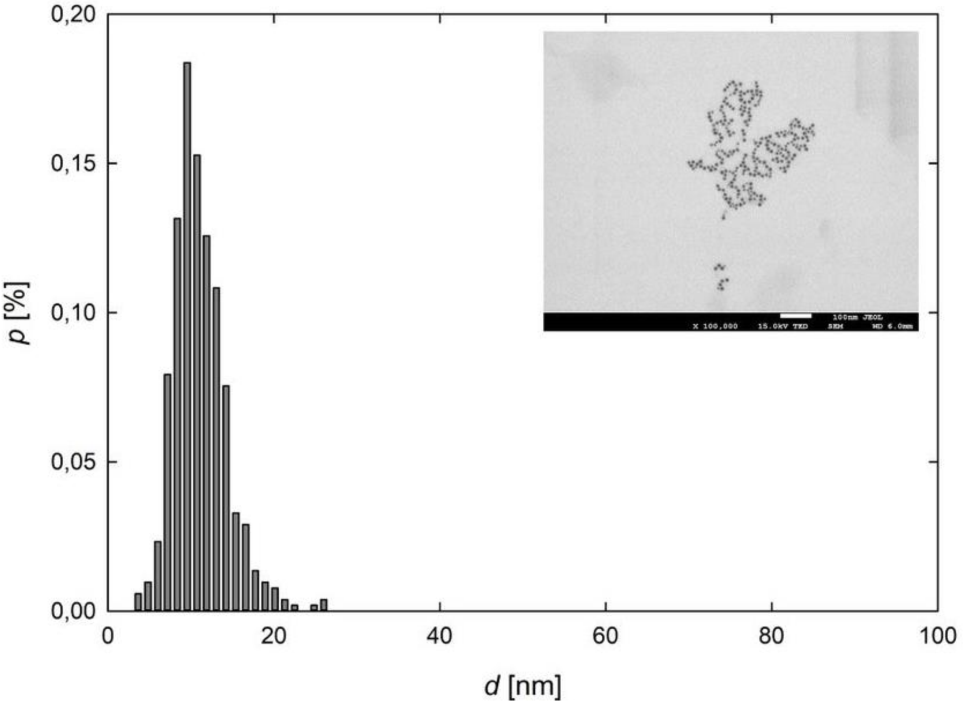
0,20
0,15
0,10
0,05
0,00
p [%]
0
20
40
60
80
100
d [nm]

(**o**)
